# Associations of concurrent early‐life famine exposure and adulthood obesity with type 2 diabetes mellitus in middle‐aged Chinese

**DOI:** 10.1111/1753-0407.13480

**Published:** 2023-10-26

**Authors:** Qian Yi, Jing Wu, Yaojia Shen, Yunying Zhu, Yiyang Zhou, He Bai, Jiajun Hao, Peige Song

**Affiliations:** ^1^ School of Public Health and Women's Hospital Zhejiang University School of Medicine, Zhejiang University Hangzhou China; ^2^ School of Public Health, Zhejiang University School of Medicine Zhejiang University Hangzhou China; ^3^ Department of Maternal and Child Health, School of Public Health Peking University Beijing China

**Keywords:** famine, obesity, overweight, type 2 diabetes mellitus

## Abstract

**Background:**

Evidence has shown that early‐life famine exposure and obesity in adulthood are independently associated with the risk of type 2 diabetes mellitus (T2DM). However, few studies had revealed the combined effect of these risk factors.

**Methods:**

Two sets of groups from the China Health and Retirement Longitudinal Study (CHARLS) were selected. The fetal‐exposure group born in 1959–1961 from 2011 wave (*N* = 958) and nonexposure group born in 1963–1965 from 2015 wave (*N* = 1540) were selected as Comparison 1. The early childhood‐exposure group born in 1955–1957 from 2011 wave (*N* = 1510) and fetal‐exposure group born in 1959–1961 from 2015 wave (*N* = 943) were Comparison 2. Logistic regressions were applied to examine the associations of different famine exposure periods and obesity patterns with T2DM risk.

**Results:**

Compared with nonexposed participants without central overweight/obesity in adulthood, central overweight/obesity in adulthood together with nonexposure (odds ratio [OR]: 1.89, 95% confidence interval [CI]: 1.19–3.00) or fetal‐exposure (OR: 1.99, 95% CI: 1.23–3.23) was associated with higher risks of T2DM. Compared with the early childhood‐exposure group, the fetal‐exposed participants showed higher risks of T2DM (OR: 1.30, 95% CI: 1.02–1.66). The coexistence of fetal famine exposure and central overweight/obesity in adulthood was associated with higher risks of T2DM (OR: 1.82, 95% CI: 1.19–2.79). Consistent associations were observed among males and participants from less severely affected areas.

**Conclusions:**

In conclusion, central overweight/obesity in adulthood is associated with the increased risk of T2DM, but the effect of early‐life famine exposure is not very clear.

## Graphical abstract

**Highlights**

- Central overweight/obesity in adulthood is associated with the increased risk of type 2 diabetes mellitus, but the effect of early‐life famine exposure is not very clear.
- The fetal period is probably a sensitive time window for the risk of type 2 diabetes mellitus, and central obesity in adulthood also plays a crucial role.
- Although lifestyle modification in adulthood will provide a degree of prevention, the long‐term benefit may also come from the improvement of early life growth and development.

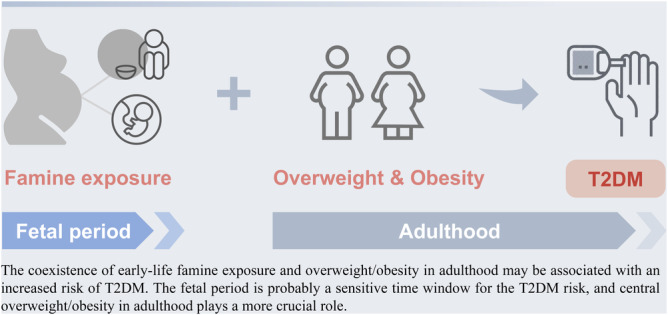

## 1 INTRODUCTION

Type 2 diabetes mellitus (T2DM) is one of the most important noncommunicable diseases with increasing prevalence, which is considered a public health problem in China and the world. By 2019, there were 437.9 million cases of T2DM globally, which has increased by 50% since 1990.^1^ The incidence rate of T2DM in the Chinese population was also found increasing from 1990 to 2019, especially among adults aged 45–54.^2^ As the burden of T2DM grows, people's quality of life will be adversely affected, bringing the increasing public health challenge and medical care needs.

The fetal origin hypothesis was first proposed in 1995, revealing that the nutrition status during the fetal period may be related to some diseases in adulthood.^3^ Afterwards, the developmental origins of health and disease hypothesis was formed. It states that early‐life experiences may profoundly affect the risk of diseases in adulthood, especially metabolic diseases.^4^ Given that ethical concerns and pathogenic risks brought by maternal nutritional intervention are hard to avoid, naturally historical events like the Chinese Great Famine (1959–1961) provide the feasibility to explore the profound impact of early‐life malnutrition. For instance, previous studies demonstrated that early‐life malnutrition due to famine may lead to a higher risk of T2DM in later life.^5^, ^6^

Otherwise, overweight and obesity in adulthood may modify the association between early‐life famine exposure and T2DM. Chinese scholars have proposed the “Two‐Hit” hypothesis of lifecycle nutritional imbalance, that is, early‐life famine exposure (malnutrition) and economic development in adulthood (overnutrition) are associated with increased risk of diabetes in adulthood.^7^ However, the extra impact of obesity phenotype has not been fully explored yet. Moreover, attention should also be paid to the unadjusted age, which may cause potential bias.

In this study, we aimed to explore the association of early‐life famine exposure and adult overweight/obesity phenotypes with the risk of T2DM in adulthood and minimize the age bias by using the data of 2011 and 2015 from the China Health and Retirement Longitudinal Study (CHARLS). Besides, we also aimed to determine the roles of the exposure period, sex, and famine severity in these associations.

## 2 METHODS

### 2.1 Study Participants

Participants in this study were from the CHARLS, a nationally representative longitudinal survey of the middle‐aged and elderly population in China.^8^ Since the baseline survey in 2011, follow‐up surveys have been conducted every 2 years. To date, the data from surveys in 2011, 2013, 2015, and 2018 have been available online (http://charls.pku.edu.cn/). Detailed information on the CHARLS has been described elsewhere.^8^ For our study, CHARLS 2011 and 2015 waves with available data related to metabolism were selected. CHARLS was approved by the Ethics Review Committee of Peking University and implemented by the National School of Development of Peking University (China Center for Economic Research). Written informed consent was obtained from each participant.

### 2.2 Famine exposure and severity

The Great Famine spread throughout the mainland of China from 1959 to 1961.^9^, ^10^ The participants were categorized into three groups according to their birthdates: (a) the nonexposed group (born between January 1, 1963 and December 31, 1965); (b) the fetal‐exposed group (born between January 1, 1959 and December 31, 1961); and (c) the early childhood‐exposed group (born between January 1, 1955 and December 31, 1957).^11^, ^12^ Consistent with previous studies, we employed the excess death rate (EDR) to assess famine severity at the province level.^13^, ^14^ The EDR was calculated as the percentage change between the average death rate for the 3 years preceding the famine (1956–1958) and the highest death rate during the famine (1959–1961).^15^ Areas with higher EDR (>100%) were considered severely affected by famine, and others were defined as less severely affected areas (Table S1).

### 2.3 Definition of obesity patterns

Body mass index (BMI) was calculated by height and weight (BMI = weight/height^2^ kg/m^2^). According to the cutoff points proposed by the Working Group on Obesity in China, general overweight/obesity was defined as BMI ≥24.0 kg/m^2^, and central overweight/obesity was defined as waist circumference (WC) ≥ 85 cm in males or ≥ 80 cm in females.^16^

### 2.4 Definition of type 2 diabetes mellitus

After blood sample collection, fasting blood glucose (FPG) was examined by enzymatic colorimetric analysis. Hemoglobin A1c (HbA1c) was tested by the High Performance Liquid Chromatography. According to the diagnostic criteria from the Chinese Diabetes Society (2021),^17^ participants who met any one of the following conditions were considered to be diabetic: (a) self‐report of the application of insulin or antidiabetic drugs; (b) FBG ≥126 mg/dL (7.0 mmol/L); (c) random blood glucose ≥200 mg/dL (11.1 mmol/L); and (d) HbA1c ≥ 6.5%.

### 2.5 Covariates

Demographic data on sex, educational status, marital status, and region were collected from the 2011 CHARLS baseline survey. Smoking status was categorized into never smoking, past smoking, or current smoking groups. Drinking status was divided into never drinking, past drinking, or current drinking groups.

### 2.6 Statistical analysis

The baseline characteristics of included participants were summarized as number (percentage) for categorical variables. Chi‐square test was used to analyze the consistency of categorical variables between groups. To explore the association between early‐life famine exposure and the risk of T2DM, logistic regression was applied to estimate odds ratios (ORs) and 95% confidence intervals (CIs). The crude model did not adjust for any covariates. Model 1 was adjusted for sex (not included when stratified by sex), marital status, region, education, and famine severity (not included when stratified by famine severity). Model 2 was additionally adjusted for smoking and drinking history. Model 3 was further adjusted for general or central obesity based on model 2.

For the combined effect analysis, a four‐subgroup categorical variable was formed based on famine exposure and obesity measures to investigate the association between the risk of T2DM and the coexistence of early‐life famine exposure with central overweight/obesity in adulthood. WC < 80/85 cm and nonexposure (or childhood exposure) were regarded as the reference in different settings. The multivariable logistic model was also adjusted as mentioned previously. Considering that the effect of early‐life famine exposure and adult obesity on T2DM risks might have sex specificity and regional imbalance, stratified analyses were conducted. The multivariable model was also adjusted for covariates. Given that different obesity phenotypes may have different impacts on the T2DM risk, a sensitivity analysis was conducted to explore the effects of general overweight or obesity.

Statistical analyses were performed with SAS (version 9.4). Two‐sided *p* value <.05 was considered significant.

## 3 RESULTS

### 3.1 Participant characteristics

**FIGURE 1:**
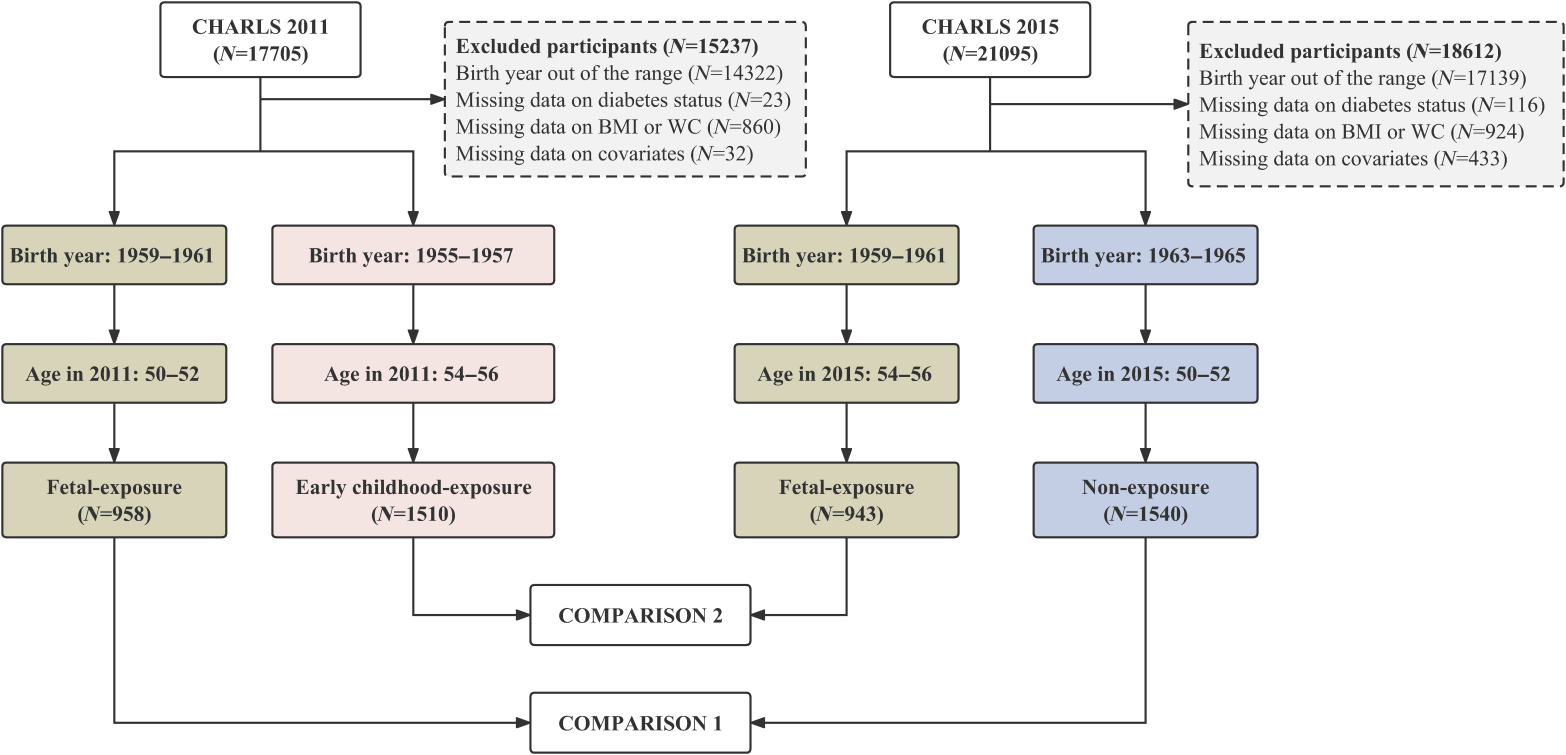
Flowchart of sample selection. BMI, body mass index; CHARLS, China Health and Retirement Longitudinal Study; WC, waist circumstance.

Participants born in 1955–1957 and 1959–1961 were selected from CHARLS 2011 (*N* = 17 705), and participants born in 1959–1961 and 1963–1965 were selected from CHARLS 2015 (*N* = 21 095). Excluding participants with unsuitable or missing data, we included the fetal‐exposure group from CHARLS 2011 (*N* = 958) and the nonexposure group from CHARLS 2015 (*N* = 1540) for Comparison 1. For Comparison 2, we included the early childhood‐exposure group from CHARLS 2011 (*N* = 1510) and the fetal‐exposure group from CHARLS 2015 (*N* = 943) (Figure 1). The characteristics of participants in two sets of comparisons are presented in Table 1.

**TABLE 1:** Baseline characteristics of participants in Comparison 1 and Comparison 2.

Characteristics	Comparison 1		Comparison 2	
	Fetal exposure (*N* = 958)	Nonexposure (*N* = 1540)	Childhood exposure (*N* = 1510)	Fetal exposure (*N* = 943)
Birth year	1959–1961	1963–1965	1955–1957	1959–1961
Age range, years	50–52	50–52	54–56	54–56
Sex, *n* (%)				
Male	429 (44.8)	643 (41.8)	722 (47.8)	409 (43.4)
Female	529 (55.2)	897 (58.2)	788 (52.2)	534 (56.6)
Education, *n* (%)				
Primary school or below	463 (48.3)	778 (50.5)	939 (62.2)	481 (51.0)
Junior school	268 (28.0)	546 (35.5)	347 (23.0)	252 (26.7)
Senior school or above	227 (23.7)	216 (14.0)	224 (14.8)	210 (22.3)
Marital status, *n* (%)				
Marriage or cohabitation	898 (93.7)	1479 (96.0)	1412 (93.5)	879 (93.2)
Others	60 (6.3)	61 (4.0)	98 (6.5)	64 (6.8)
Region, *n* (%)				
Rural	594 (62.0)	950 (61.7)	974 (64.5)	607 (64.4)
Urban	364 (36.4)	590 (38.3)	536 (35.5)	336 (35.6)
Smoking, *n* (%)				
Never	609 (63.6)	1008 (65.5)	872 (57.7)	580 (61.5)
Past or current	349 (36.4)	532 (34.5)	638 (42.3)	363 (38.5)
Drinking, *n* (%)				
Never	674 (70.4)	1044 (67.8)	1012 (67.0)	636 (67.4)
Past or current	284 (29.6)	496 (32.2)	498 (33.0)	307 (32.6)
Famine severity, *n* (%)				
Severely affected area	327 (34.1)	‐	603 (39.9)	323 (34.3)
Less severely affected area	631 (65.9)	‐	907 (60.1)	620 (65.7)
General obesity, *n* (%)				
No	504 (52.6)	694 (45.1)	877 (58.1)	461 (48.9)
Yes	454 (47.4)	846 (54.9)	633 (41.9)	482 (51.1)
Central obesity, *n* (%)				
No	358 (37.4)	479 (31.1)	612 (40.5)	301 (31.9)
Yes	600 (62.6)	1061 (68.9)	898 (59.5)	642 (68.1)
Type 2 diabetes, *n* (%)			
No	849 (88.6)	1354 (87.9)	1336 (88.5)	807 (85.6)
Yes	109 (11.4)	186 (12.1)	174 (11.5)	136 (14.4)

*Note*: Data were presented as number (*N*) with percent (%). For famine severity, we employed the excess death rate (EDR) to assess famine severity at the province level. The EDR was calculated as the percentage change between the average death rate for the 3 years preceding the famine (1956–1958) and the highest death rate during the famine (1959–1961). Areas with higher EDR (>100%) were considered severely affected by famine, and others were defined as less severely affected areas.

Participants in Comparison 1 were aged from 50 to 52 in both groups. Compared with the nonexposed group, participants with fetal exposure were more educated, more likely to be single, and had a lower prevalence of both general and central overweight or obesity. Participants in Comparison 2 were aged from 54 to 56 in both groups. Compared to the early childhood‐exposure group, the fetal‐exposure group had a slightly higher proportion of female participants. Moreover, participants with fetal exposure were more educated, experienced less severe famine, and had a higher prevalence of both general and central overweight or obesity.

### 3.2 Individual association of fetal famine exposure with T2DM

The associations between famine exposure and T2DM are reported in Table S2. In Comparison 1, the prevalence of T2DM was 11.4% (109 cases) in the fetal‐exposure group and 12.1% (186 cases) in the nonexposure group. Compared with unexposed participants, those with fetal exposure did not demonstrate increased risks of T2DM in any model. In Comparison 2, 14.4% (136 cases) of participants in the fetal‐exposure group were diagnosed with T2DM. This was significantly higher than the 11.5% (174 cases) in the early childhood‐exposure group. We also found that participants with fetal exposure had higher risks of T2DM than those with early childhood exposure, with a crude OR of 1.29 (95% CI 1.02–1.65). This significant association remained in both model 1 (OR = 1.30, 95% CI 1.02–1.66) and model 2 (OR = 1.30, 95% CI 1.02–1.66). However, after incorporating overweight/obesity phenotypes into model 3, this association was no longer significant (OR = 1.22, 95% CI 0.96–1.57). To explore the impacts of sex, famine severity, and overweight/obesity phenotypes on the association between famine exposure and T2DM, we further conducted stratified analyses. *p* values for interaction are provided in Tables S2 and S3.

### 3.3 Association of concurrent famine exposure and adulthood overweight/obesity with T2DM

The associations of concurrent famine exposure and central overweight/obesity with T2DM are shown in Tables 2 and 3. In Comparison 1, central overweight/obesity alone (OR for central overweight/obesity and nonfamine exposure = 1.89, 95% CI 1.19–3.00) or in conjunction with fetal‐exposure (OR for central overweight/obesity and fetal‐exposure = 1.99, 95% CI 1.23–3.23) was associated with a higher T2DM risk, using nonexposed participants without central overweight/obesity as a reference. In Comparison 2, the reference consisted of early childhood‐exposure participants without central overweight/obesity. The findings indicated that fetal famine exposure, along with central overweight or obesity, was associated with higher risks of T2DM (OR = 1.82, 95% CI 1.19–2.79).

**TABLE 2:** Associations of concurrent famine exposure and central obesity with T2DM in Comparison 1.

	Nonexposure		Fetal exposure	
	OR (95% CI)		OR (95% CI)	
	Normal weight	Central obesity	Normal weight	Central obesity
Total				
Crude model	1.00	2.47 (1.65–3.70)	0.86 (0.48–1.53)	2.52 (1.64–3.86)
Model 1	1.00	2.56 (1.71–3.85)	0.87 (0.49–1.56)	2.68 (1.73–4.13)
Model 2	1.00	2.59 (1.72–3.88)	0.88 (0.49–1.57)	2.70 (1.75–4.17)
Model 3	1.00	1.89 (1.19–3.00)	0.89 (0.50–1.60)	1.99 (1.23–3.23)
Male				
Crude model	1.00	3.56 (1.99–6.36)	0.80 (0.35–1.82)	3.22 (1.72–6.05)
Model 1	1.00	3.57 (1.99–6.42)	0.79 (0.35–1.81)	3.29 (1.73–6.24)
Model 2	1.00	3.61 (2.00–6.49)	0.81 (0.35–1.85)	3.34 (1.76–6.35)
Model 3	1.00	2.14 (1.05–4.39)	0.83 (0.36–1.91)	2.12 (1.01–4.43)
Female				
Crude model	1.00	1.87 (1.07–3.26)	0.94 (0.41–2.13)	2.07 (1.15–3.72)
Model 1	1.00	1.85 (1.06–3.24)	1.02 (0.45–2.31)	2.15 (1.19–3.87)
Model 2	1.00	1.85 (1.06–3.24)	1.01 (0.45–2.31)	2.14 (1.19–3.85)
Model 3	1.00	1.55 (0.84–2.85)	1.01 (0.44–2.31)	1.77 (0.93–3.35)
*p* for interaction				.2742
Less severely affected area				
Crude model	1.00	2.63 (1.56–4.43)	0.76 (0.36–1.62)	2.86 (1.65–4.94)
Model 1	1.00	2.74 (1.61–4.64)	0.81 (0.38–1.72)	3.07 (1.76–5.35)
Model 2	1.00	2.75 (1.62–4.67)	0.81 (0.38–1.72)	3.08 (1.77–5.38)
Model 3	1.00	1.99 (1.10–3.60)	0.83 (0.39–1.76)	2.24 (1.21–4.15)
Severely affected area				
Crude model	1.00	2.23 (1.18–4.19)	1.05 (0.42–2.61)	1.93 (0.96–3.90)
Model 1	1.00	2.36 (1.24–4.46)	0.99 (0.40–2.48)	1.99 (0.97–4.05)
Model 2	1.00	2.38 (1.26–4.52)	1.01 (0.40–2.53)	2.01 (0.98–4.10)
Model 3	1.00	1.85 (0.88–3.87)	1.04 (0.41–2.62)	1.60 (0.73–3.52)
*p* for interaction				.6105

*Note*: Associations were expressed as odds ratio with 95% CI. Central obesity was defined as WC ≥80 cm for females and WC ≥85 cm for males. Model 1 was adjusted for sex (not included if it was used as a stratification index), marital status, region, education, and famine severity (not included if it was used as a stratification index). Model 2 was additionally adjusted for smoking and drinking history. Model 3 was further adjusted for general obesity based on model 2.

Abbreviations: CI, confidence interval; OR, odds ratio; T2DM, type 2 diabetes mellitus; WC, waist circumference.

**TABLE 3:** Associations of concurrent famine exposure and central obesity with T2DM in Comparison 2.

	Childhood‐exposure		Fetal‐exposure	
	OR (95% CI)		OR (95% CI)	
	Normal weight	Central obesity	Normal weight	Central obesity
Total				
Crude model	1.00	1.92 (1.35–2.72)	0.79 (0.46–1.37)	2.62 (1.83–3.74)
Model 1	1.00	1.79 (1.25–2.56)	0.78 (0.45–1.36)	2.49 (1.73–3.60)
Model 2	1.00	1.79 (1.25–2.57)	0.78 (0.45–1.36)	2.50 (1.73–3.61)
Model 3	1.00	1.32 (0.87–2.00)	0.79 (0.45–1.37)	1.82 (1.19–2.79)
Male				
Crude model	1.00	2.07 (1.26–3.41)	0.62 (0.28–1.39)	2.95 (1.76–4.93)
Model 1	1.00	2.00 (1.20–3.33)	0.62 (0.27–1.39)	2.91 (1.72–4.93)
Model 2	1.00	2.00 (1.20–3.33)	0.62 (0.28–1.40)	2.89 (1.71–4.90)
Model 3	1.00	1.31 (0.69–2.48)	0.63 (0.28–1.42)	1.93 (1.00–3.71)
Female				
Crude model	1.00	1.65 (1.00–2.75)	0.98 (0.46–2.11)	2.20 (1.32–3.69)
Model 1	1.00	1.58 (0.95–2.63)	0.96 (0.44–2.06)	2.16 (1.29–3.62)
Model 2	1.00	1.57 (0.94–2.61)	0.99 (0.46–2.13)	2.18 (1.30–3.66)
Model 3	1.00	1.25 (0.71–2.19)	0.99 (0.46–2.14)	1.70 (0.96–3.04)
*p* for interaction				.5451
Less severely affected area				
Crude model	1.00	2.28 (1.37–3.78)	1.10 (0.54–2.26)	3.70 (2.24–6.12)
Model 1	1.00	2.10 (1.25–3.52)	1.07 (0.52–2.20)	3.40 (2.03–5.70)
Model 2	1.00	2.10 (1.25–3.53)	1.07 (0.52–2.19)	3.44 (2.05–5.77)
Model 3	1.00	1.48 (0.82–2.66)	1.05 (0.51–2.16)	2.37 (1.31–4.28)
Severely affected area				
Crude model	1.00	1.69 (1.03–2.76)	0.54 (0.21–1.34)	1.72 (1.01–2.94)
Model 1	1.00	1.64 (0.98–2.72)	0.55 (0.22–1.38)	1.75 (1.00–3.05)
Model 2	1.00	1.65 (0.99–2.75)	0.54 (0.22–1.37)	1.73 (0.99–3.03)
Model 3	1.00	1.32 (0.73–2.41)	0.55 (0.22–1.37)	1.37 (0.72–2.62)
*p* for interaction				.1863

*Note*: Associations were expressed as odds ratio with 95% CI. Central obesity was defined as WC ≥80 cm for females and WC ≥85 cm for males. The crude model did not adjust for any covariates. Model 1 was adjusted for sex (not included if it was used as a stratification index), marital status, region, education, and famine severity (not included if it was used as a stratification index). Model 2 was additionally adjusted for smoking and drinking history. Model 3 was further adjusted for general obesity based on model 2.

Abbreviations: CI, confidence interval; OR, odds ratio; T2DM, type 2 diabetes mellitus; WC, waist circumference.

### 3.4 Stratified analysis by sex, severity of famine, and overweight/obesity phenotypes

In Comparison 1, the combined effects of famine exposure and central overweight/obesity in adulthood were observed among males and participants from less severely affected areas (Table 2). Whether there was fetal famine exposure or not, central overweight/obesity might result in an increased risk of T2DM in males (nonexposure: OR = 2.14, 95% CI 1.05–4.39; fetal exposure: OR = 2.12, 95% CI 1.01–4.43) or participants from less severely affected areas (nonexposure: OR = 1.99, 95% CI 1.10–3.60; fetal exposure: OR = 2.24, 95% CI 1.21–4.15).

The famine severity and sex‐specific results of Comparison 2 were consistent with Comparison 1 (Table 3). After stratification by sex and famine severity, associations similar to those in the total population were observed only in males (OR = 1.93, 95% CI 1.00–3.71) or participants from less severely affected areas (OR = 2.37, 95% CI 1.31–4.28).

### 3.5 Sensitivity analysis

In addition to central overweight/obesity, general overweight/obesity was taken into consideration in the sensitivity analysis. In Comparison 1, we found that the coexistence of general overweight/obesity and fetal exposure (OR = 1.62, 95% CI 1.06–2.47) or nonexposure (OR = 1.55, 95% CI 1.06–2.28) was associated with an increased risk of T2DM. Similar associations were observed in participants from less severely affected areas (OR for general overweight/obesity and nonexposure = 1.64, 95% CI 1.02–2.66; OR for general overweight/obesity and fetal exposure = 1.75, 95% CI 1.04–2.94). In Comparison 2, the combined effect of fetal exposure and general overweight/obesity on the increased risk of T2DM was found to be significant in the total population (OR = 1.96, 95% CI 1.35–2.86), subgroups of males (OR = 2.16, 95% CI 1.14–4.08), females (OR = 1.92, 95% CI 1.20–3.07), and less severely affected areas (OR = 2.53, 95% CI 1.52–4.21). For participants living in severely affected area, those with normal weight and fetal exposure were associated with a lower risk of T2DM, with a fully adjusted OR of 0.47 (95% CI 0.23–0.94, see Tables S4 and S5).

The birth month of the participants may influence the group to which they were assigned. Therefore, we conducted a sensitivity analysis excluding individuals born in 1959 and 1961 from the exposure group. In the total population of Comparison 1, we found that central overweight/obesity was associated with an increased risk of T2DM whether coexisted with the fetal‐exposure (OR = 2.10, 95% CI 1.24–3.57) or not (OR = 1.95, 95% CI 1.21–3.14). In Comparison 2, we identified a significant combined association of fetal exposure and central overweight/obesity with the increased risk of T2DM in the total population (OR = 1.98, 95% CI 1.25–3.15) (Tables S6 and S7).

## 4 DISCUSSION

Based on the occurrence period of the Chinese famine, this study selected participants from CHARLS 2011 and 2015 to form two sets of comparisons, thereby making the ages more comparable for the analyses. Compared to participants exposed to famine in early childhood, those exposed in the fetal stage demonstrated higher risks of T2DM among participants aged 54–56 (Comparison 2). Furthermore, the concurrent presence of fetal exposure and central overweight or obesity in adulthood posed higher risks of T2DM. After stratification by sex and famine severity, this association was observed only in males and participants from less severely affected areas. When the nonexposed group served as the reference, there was no significant association between fetal famine exposure and T2DM among participants aged 50–52 (Comparison 1). Nevertheless, we found that central overweight/obesity in adulthood, regardless of concurrent fetal famine exposure, increased the risk of T2DM in the total population, subgroups of males, and participants from less severely affected areas.

Previous studies have concluded that an association exists between early‐life famine exposure and an increased risk of T2DM. For instance, Ravelli et al suggested that prenatal exposure to the Dutch famine was linked to an increased risk of T2DM.^18^ Based on the study of the Chinese famine, Wang et al concluded that exposure to famine during childhood led to a higher risk of T2DM and hyperglycemia in adulthood.^19^ Conversely, our findings were not entirely consistent with previous studies, as we did not observe a significant difference between the non‐exposed and fetal‐exposed groups. We only found that fetal‐exposed participants have a higher risk of T2DM than those exposed to famine during early childhood. One possible explanation for this discrepancy is that factors such as age at famine exposure, sex, race, smoking status, dietary patterns, and use of antilipemic medications among the included participants may modify the association between famine and T2DM events. Another possible explanation for the inconsistent results across studies is the differing diagnostic criteria for T2DM used in various studies. Our study employed the Chinese Diabetes Society (2021) criteria, whereas Zhang et al^20^ adopted the World Health Organization and International Diabetes Federation 1999 criteria. Additionally, the inconsistent results could be attributed to differences in the timing of famine exposure during fetal development. A study of the Ukraine famine by Lumey et al suggested that early gestation may be a crucial timing window for determining the risk of T2DM.^5^ However, our study was unable to distinguish the effect of various fetal stages, as the precise timing of exposure has not yet been determined. Particularly, several previous studies, such as Wang et al,^19^ did not control for age between groups or consider the potential aging effects, which made it incomparable to our study. More specifically, some participants exposed to famine in early childhood in these previous studies might have also experienced famine during the fetal period. In addition, the younger age compared to Comparison 2 and the limited sample sizes may contribute to the insignificant results.

Although previous studies have proposed the hypothesis that poor early growth conditions may lead to physiological adaptations,^4^ the mechanisms of these associations remain unclear. The studies on the epigenetic mechanism have gained popularity in recent years, promoting the rise of thrifty epigenotype hypothesis. For instance, differential methylation patterns like insulin signaling and pancreatic beta cell functioning (SMAD7) have been found effective in the epigenetic control of metabolic processes after exposure to malnutrition, and the epigenetic modifications like microRNA have also been found to be influential.^4^, ^21^ Additionally, the epigenetic modifications accumulate with age and ultimately lead to metabolic diseases in adulthood, which were even considered to have intergenerational heritable potential.^22^

Consistent with some previous studies, we found that increased risk of T2DM associated with early‐life famine exposure may be exacerbated by obesity in adulthood. Ravelli et al emphasized that the effect of early‐life famine exposure on glucose tolerance is more pronounced in individuals who become obese during adulthood,^18^ and Meng et al concluded that the coexistence of fetal malnutrition and central obesity in adulthood leads to a higher risk of T2DM.^6^ Drawing from the results of Survey on the Prevalence in East China for Metabolic Diseases and Risk Factors (SPECT‐China), Wang et al found that the increased prevalence of T2DM may be related to the combination of early‐life famine exposure and improved nutrition in adulthood.^7^

Exploration of the mechanisms behind these findings mainly focuses on the nutritional transition, in other words, the“Two‐Hit” hypothesis of lifecycle nutritional imbalance. Li et al suggested that the relationship between fetal famine exposure and the higher risk of hyperglycemia in adulthood appeared to be strengthened by the nutritional transition, such as improved economic status or a Western diet pattern, indicating a failure to match nutritional patterns.^23^ As the interaction between prenatal and postnatal environment has been shown to affect the risk of noncommunicable diseases like T2DM, many studies have shown that physiological adaptations stemming from poor growth may increase disease risk under enriched conditions.^4^ Moreover, Wang et al delved into the epigenetic changes and found that the expression of miR‐128‐1 microRNA might alter due to poor early‐life conditions, potentially leading to metabolic maladaptation with the improved nutrition.^24^ Wang et al proposed the “Two‐Hit” hypothesis of lifecycle nutritional imbalance, that is, famine in early life and rapid economic development in adulthood may further increase the risk of T2DM. People who experience both malnutrition in early life and overnutrition in adulthood suffer from decreased lipid storage and metabolic capacity, setting off severe ectopic fat deposition and resulting in nonobese insulin resistance, which is usually manifested as central obesity.^7^

Conversely, we also observed the aforementioned associations in the unexposed group. In the less severely affected areas, the results of Comparison 2 were consistent with the findings in the total population, and Comparison 1 showed a similar association. Among participants from areas severely affected by famine, only the association of fetal famine exposure with a lower risk of T2DM was found among participants without general overweight/obesity according to BMI (Comparison 2, aged 54–56). For participants aged 49–52 (Comparison 1), no significant association was found between fetal famine exposure and T2DM compared to the nonfamine exposed group. In contrast to our study, Li et al found that famine severity was positively correlated with the risk of T2DM,^23^ and Lumey et al also observed a dose–response relationship.^5^ The discrepancy may be due to different classification criteria for famine severity and the limited sample size after stratification. Moreover, it is plausible that survivors of severe famine may possess superior genetic traits and a lower risk of T2DM, an effect of natural selection brought about by the famine.^25^, ^26^

Furthermore, the association was observed only in males, which was inconsistent with some previous studies. For example, Sun et al found that the association between early‐life famine exposure and T2DM risk was more robust in females.^27^ Several potential reasons could explain this discrepancy. First, males may be more susceptible to malnutrition during the fetal period, as DNA methylation may be sex specific.^7^, ^28^ Moreover, in the Chinese population, the proportion of males who develop central obesity in middle age is higher than that of females who tend to develop general obesity in old age.^29^ Additionally, differences in fat accumulation and hormone levels due to sex could potentially have an impact, along with the disparities introduced by publication bias.

This study boasts several strengths. First, given the limited extrapolation of animal experiments and ethical problems of human experiments, this study focused on the Chinese famine, a natural event that occurred roughly from 1959 to 1961. Moreover, the Chinese famine's extended duration and its impact on a vast number of people across large areas of China provided suitable conditions for study. Second, our study was based on a representative sample from CHARLS, utilizing high‐quality data. Third, as few studies have focused on the combined associations of early‐life famine exposure and adult overweight/obesity with the risk of T2DM, our study explored and validated the “Two Hit” hypothesis of lifecycle nutritional imbalance. Lastly, the two sets of comparisons formed in this study were selected from CHARLS 2011 and 2015 to ensure age comparability and avert bias stemming from age imbalances between groups and the aging effect.

However, our study also had the following limitations. First, due to the vague start and end times of the Chinese famine, we were unable to classify the participants precisely according to birthdates, which could lead to potential misclassification. Second, although our study covered as many confounding factors as possible, there were other factors not included, such as variables reflecting diet, income, physical activity, and so forth. Third, the definition of famine severity remains controversial, as previous studies have applied cohort size shrinkage indices.^30^ Moreover, although this study controlled for age between groups, it could not adjust for the potential influence of the time interval between the two cross‐sectional studies. Finally, the limited sample size also resulted in reduced statistical efficiency. Therefore, future national and even international studies with larger sample sizes are needed.

In conclusion, overweight/obesity in adulthood is associated with an increased risk of T2DM, but the effect of early‐life famine exposure remains unclear. Although previous evidence suggested that the fetal period may be a sensitive window for shaping long‐term effects, our findings indicate that central obesity in adulthood plays a crucial role. More research is needed to determine whether early life famine exposure combined with adult obesity is associated with higher risks of T2DM.

## AUTHOR CONTRIBUTIONS

Peige Song designed the study. Qian Yi and Yaojia Shen managed and analyzed the data. Jing Wu and Yaojia Shen prepared the first draft. All authors were involved in revising the manuscript and approved the final version of the manuscript.

## DISCLOSURE

The authors declared that there were no conflicts of interest concerning this manuscript.

## Supporting information


**Data S1.** Supporting Information.

## Data Availability

The databases of the CHARLS are open to researchers in need and available at the following website: https://charls.pku.edu.cn/.
