# Supplementary material for: Associations of concurrent early‐life famine exposure and adulthood obesity with type 2 diabetes mellitus in middle‐aged Chinese
Source: J Diabetes. 2023 Oct 26;16(2):e13480. doi: 10.1111/1753-0407.13480 (PMC10859315; doi:10.1111/1753-0407.13480)
Supplement: Supplementary file 1 — Data S1. Supporting Information. [file JDB-16-e13480-s001.docx]

**Associations of concurrent early-life famine exposure and adulthood obesity with type 2 diabetes mellitus in middle-aged Chinese**

**Table S1.** Mortality rates (%) and excess mortality rates (%) in all provinces in 1955-1961.

Notes: The mortality rate data from 1956 to 1961 were obtained from the National Bureau of Statistics of China. EDR, excess mortality rates, were calculated as the percentage change between the average death rate for the three years preceding the famine (1956–1958) and the highest death rate during the famine (1959–1961).

**Table S2.** Association of fetal exposure to famine with T2DM in Comparison 1 and Comparison 2 [*OR (95% CI)*].

Notes: OR, odds ratio; CI, confidence interval; T2DM, type 2 diabetes mellitus. Model 1 was adjusted for sex (not included if it was used as a stratification index), marital status, region, education and famine severity (not included if it was used as a stratification index). Model 2 was further adjusted for smoking and drinking based on model 1. Model 3 was further adjusted for general obesity and central obesity based on model 2.

**Table S3.** Association of Famine exposure with T2DM according to obesity status in Comparison 1 and Comparison 2.

Note: OR, odds ratio; CI, confidence interval; BMI, Body Mass Index; WC, Waist Circumference. General obesity was defined as BMI ≥ 24.0 kg/m^2^. Central obesity was defined as WC ≥ 80cm for females and WC ≥ 85cm for males. OR values were adjusted for age, sex (not included if it was used as a stratification index), education, marital status, region, smoking, drinking, famine severity (not included if it was used as a stratification index) and general obesity (when according to WC) or central obesity (when according to BMI).

**Table S4.** Association of concurrent famine exposure, general overweight and obesity with T2DM in Comparison 1.

Note: OR, odds ratio; CI, confidence interval; Associations were expressed as odds ratio with 95% confidence interval; According to the cut-off points proposed by the Working Group on Obesity in China, general overweight/obesity was defined as BMI ≥ 24.0 kg/m2. Model 1 was adjusted for sex (not included if it was used as a stratification index), marital status, region, education, and famine severity (not included if it was used as a stratification index). Model 2 was additionally adjusted for smoking and drinking history. Model 3 was further adjusted for central obesity based on model 2.

**Table S5.** Association of concurrent famine exposure, general overweight and obesity with T2DM in Comparison 2.

Note: OR, odds ratio; CI, confidence interval; Associations were expressed as odds ratio with 95% confidence interval; According to the cut-off points proposed by the Working Group on Obesity in China, general overweight/obesity was defined as BMI ≥ 24.0 kg/m2. Model 1 was adjusted for sex (not included if it was used as a stratification index), marital status, region, education, and famine severity (not included if it was used as a stratification index). Model 2 was additionally adjusted for smoking and drinking history. Model 3 was further adjusted for central obesity based on model 2.

**Table S6.** Associations of concurrent famine exposure and central obesity with T2DM after excluding participants born in 1959 and 1961 from exposure group in Comparison 1.

Note: OR, odds ratio; CI, confidence interval; Associations were expressed as odds ratio with 95% confidence interval; According to the cut-off points proposed by the Working Group on Obesity in China, central overweight/obesity was defined as waist circumference (WC) ≥ 85 cm in males or ≥ 80 cm in females. Model 1 was adjusted for sex (not included if it was used as a stratification index), marital status, region, education, and famine severity (not included if it was used as a stratification index). Model 2 was additionally adjusted for smoking and drinking history. Model 3 was further adjusted for general obesity based on model 2.

**Table S7.** Associations of concurrent famine exposure and central obesity with T2DM after excluding participants born in 1959 and 1961 from exposure group in Comparison 2.

Note: OR, odds ratio; CI, confidence interval; Associations were expressed as odds ratio with 95% confidence interval; According to the cut-off points proposed by the Working Group on Obesity in China, central overweight/obesity was defined as waist circumference (WC) ≥ 85 cm in males or ≥ 80 cm in females. Model 1 was adjusted for sex (not included if it was used as a stratification index), marital status, region, education, and famine severity (not included if it was used as a stratification index). Model 2 was additionally adjusted for smoking and drinking history. Model 3 was further adjusted for general obesity based on model 2.

**Table S1.** Mortality rates (%) and excess mortality rates (%) in all provinces in 1955-1961.

|  | 1956 | 1957 | 1958 | 1959 | 1960 | 1961 | EDR |
| --- | --- | --- | --- | --- | --- | --- | --- |
| Beijing | 7.7 | 8.2 | 8.1 | 9.6 | 9.2 | 10.8 | 35.0 |
| Tianjin | 8.8 | 9.4 | 8.7 | 9.9 | 10.3 | 9.9 | 14.9 |
| Hebei | 11.3 | 11.3 | 10.3 | 12.3 | 15.8 | 13.6 | 44.1 |
| Shanxi | 11.6 | 12.7 | 11.7 | 12.8 | 14.2 | 12.2 | 18.3 |
| Inner Mongoria | 7.9 | 10.5 | 7.9 | 11.0 | 9.5 | 8.8 | 25.5 |
| Liaoning | 6.6 | 9.4 | 8.8 | 11.8 | 11.5 | 17.5 | 111.7 |
| Jilin | 7.5 | 9.1 | 9.1 | 13.4 | 10.1 | 12.1 | 56.4 |
| Heilongjiang | 10.1 | 10.5 | 9.2 | 12.8 | 10.5 | 11.1 | 28.9 |
| Shanghai | 6.8 | 6.0 | 5.9 | 6.9 | 6.8 | 7.7 | 23.5 |
| Jiangsu | 13.0 | 10.3 | 9.4 | 14.6 | 18.4 | 13.4 | 68.8 |
| Zhejiang | 9.5 | 9.3 | 9.2 | 10.8 | 11.9 | 9.8 | 27.5 |
| Anhui | 14.3 | 9.1 | 12.4 | 16.7 | 68.6 | 8.1 | 474.9 |
| Fujian | 8.4 | 7.9 | 7.5 | 7.9 | 15.3 | 11.9 | 92.9 |
| Jiangxi | 12.5 | 11.5 | 11.3 | 13 | 16.1 | 11.5 | 36.8 |
| Shandong | 12.1 | 12.1 | 12.8 | 18.2 | 23.6 | 18.5 | 91.4 |
| Henan | 14.0 | 11.8 | 12.7 | 14.1 | 39.6 | 10.2 | 208.6 |
| Hubei | 10.9 | 9.6 | 9.6 | 14.5 | 21.2 | 9.2 | 111.3 |
| Hunan | 11.5 | 10.4 | 11.7 | 13.0 | 29.4 | 17.5 | 162.5 |
| Guangdong | 11.2 | 8.4 | 9.1 | 11.7 | 15.1 | 10.7 | 57.8 |
| Guangxi | 12.5 | 12.4 | 11.7 | 17.5 | 29.5 | 19.5 | 141.8 |
| Sichuan | 10.4 | 12.1 | 25.2 | 47.0 | 54.0 | 29.4 | 239.6 |
| Guizhou | 13.0 | 12.4 | 15.3 | 20.3 | 52.3 | 23.3 | 285.5 |
| Yunnan | 15.2 | 16.3 | 21.6 | 18.0 | 26.3 | 11.9 | 48.6 |
| Shaanxi | 9.9 | 10.3 | 11.0 | 12.7 | 12.3 | 8.7 | 22.1 |
| Gansu | 10.8 | 11.3 | 21.1 | 17.4 | 41.3 | 11.5 | 186.8 |
| Qinghai | 9.4 | 10.4 | 13.0 | 16.6 | 40.7 | 11.7 | 272.3 |
| Ningxia | 10.6 | 11.1 | 15.0 | 15.8 | 13.9 | 10.7 | 29.2 |
| Xinjiang Uyghur  Autonomous Region | 13.9 | 13.9 | 13.9 | 18.8 | — | — | 35.3 |
| China | 11.4 | 10.8 | 12.0 | 14.6 | 25.4 | 14.2 | 122.8 |

Notes: The mortality rate data from 1956 to 1961 were obtained from the National Bureau of Statistics of China. EDR, excess mortality rates, were calculated as the percentage change between the average death rate for the three years preceding the famine (1956–1958) and the highest death rate during the famine (1959–1961).

**Table S2.** Association of fetal exposure to famine with T2DM in Comparison 1 and Comparison 2 [*OR (95% CI)*].

|  |  | Comparison 1 | | Comparison 2 | |  |
| --- | --- | --- | --- | --- | --- | --- |
|  |  | Non-exposure | Fetal-exposure | Childhood-exposure | Fetal-exposure |  |
| Total | |  |  |  |  |  |
|  | No. of cases | 186 | 109 | 174 | 136 |  |
|  | Crude model | 1.00 | 0.94 (0.73, 1.20) | 1.00 | 1.29 (1.02, 1.65) |  |
|  | Model 1 | 1.00 | 0.95 (0.74, 1.23) | 1.00 | 1.30 (1.02, 1.66) |  |
|  | Model 2 | 1.00 | 0.95 (0.74, 1.23) | 1.00 | 1.30 (1.02, 1.66) |  |
|  | Model 3 | 1.00 | 1.02 (0.79, 1.32) | 1.00 | 1.22 (0.96, 1.57) |  |
| Male | |  |  |  |  |  |
|  | No. of cases | 86 | 47 | 73 | 50 |  |
|  | Crude model | 1.00 | 0.80 (0.55, 1.16) | 1.00 | 1.24 (0.85, 1.82) |  |
|  | Model 1 | 1.00 | 0.78 (0.53, 1.15) | 1.00 | 1.25 (0.85, 1.84) |  |
|  | Model 2 | 1.00 | 0.80 (0.54, 1.17) | 1.00 | 1.25 (0.85, 1.85) |  |
|  | Model 3 | 1.00 | 0.95 (0.64, 1.42) | 1.00 | 1.17 (0.79, 1.74) |  |
| Female | |  |  |  |  |  |
|  | No. of cases | 100 | 62 | 101 | 86 |  |
|  | Crude model | 1.00 | 1.06 (0.76, 1.48) | 1.00 | 1.31 (0.96, 1.78) |  |
|  | Model 1 | 1.00 | 1.11 (0.79, 1.57) | 1.00 | 1.33 (0.97, 1.82) |  |
|  | Model 2 | 1.00 | 1.11 (0.79, 1.56) | 1.00 | 1.36 (0.99, 1.87) |  |
|  | Model 3 | 1.00 | 1.12 (0.79, 1.58) | 1.00 | 1.29 (0.94, 1.78) |  |
| Less Severely affected area | | |  |  |  |  |
|  | No. of cases | 119 | 76 | 92 | 94 |  |
|  | Crude model | 1.00 | 0.95 (0.70, 1.29) | 1.00 | 1.58 (1.16, 2.15) |  |
|  | Model 1 | 1.00 | 0.98 (0.72, 1.34) | 1.00 | 1.55 (1.13, 2.12) |  |
|  | Model 2 | 1.00 | 0.99 (0.72, 1.34) | 1.00 | 1.55 (1.14, 2.13) |  |
|  | Model 3 | 1.00 | 1.07 (0.78, 1.47) | 1.00 | 1.47 (1.07, 2.02) |  |
| Severely affected area | | |  |  |  |  |
|  | No. of cases | 67 | 33 | 82 | 42 |  |
|  | Crude model | 1.00 | 0.89 (0.58, 1.39) | 1.00 | 0.95 (0.64, 1.42) |  |
|  | Model 1 | 1.00 | 0.86 (0.55, 1.35) | 1.00 | 0.97 (0.65, 1.46) |  |
|  | Model 2 | 1.00 | 0.87 (0.55, 1.36) | 1.00 | 0.96 (0.64, 1.44) |  |
|  | Model 3 | 1.00 | 0.90 (0.57, 1.42) | 1.00 | 0.90 (0.60, 1.36) |  |

Notes: OR, odds ratio; CI, confidence interval; T2DM, type 2 diabetes mellitus. Model 1 was adjusted for sex (not included if it was used as a stratification index), marital status, region, education and famine severity (not included if it was used as a stratification index). Model 2 was further adjusted for smoking and drinking based on model 1. Model 3 was further adjusted for general obesity and central obesity based on model 2.

**Table S3.** Association of Famine exposure with T2DM according to obesity status in Comparison 1 and Comparison 2.

|  |  | Comparison 1  OR (95% CI) | | Comparison 2  OR (95% CI) | |  |
| --- | --- | --- | --- | --- | --- | --- |
|  |  | Non-exposure | Fetal-exposure | Childhood-exposure | Fetal-exposure |  |
| According to BMI | | | | | |  |
|  | Normal weight | 1.00 | 1.02 (0.65, 1.60) | 1.00 | 0.94 (0.62, 1.42) |  |
|  | General obesity | 1.00 | 1.04 (0.76, 1.43) | 1.00 | 1.43 (1.04, 1.96) |  |
|  | *P* for interaction | 0.831 | | 0.082 | |  |
| According to WC | | | | | |  |
|  | Normal weight | 1.00 | 0.91 (0.50, 1.66) | 1.00 | 0.83 (0.47, 1.45) |  |
|  | Central obesity | 1.00 | 1.05 (0.79, 1.40) | 1.00 | 1.37 (1.04, 1.82) |  |
|  | *P* for interaction | 0.609 | | 0.073 | |  |

Note: OR, odds ratio; CI, confidence interval; BMI, Body Mass Index; WC, Waist Circumference. General obesity was defined as BMI ≥ 24.0 kg/m^2^. Central obesity was defined as WC ≥ 80cm for females and WC ≥ 85cm for males. OR values were adjusted for age, sex (not included if it was used as a stratification index), education, marital status, region, smoking, drinking, famine severity (not included if it was used as a stratification index) and general obesity (when according to WC) or central obesity (when according to BMI).

**Table S4.** Associations of concurrent famine exposure and general obesity with T2DM in Comparison 1.

|  |  | Non-exposure  OR (95% CI) | | Fetal-exposure  OR (95% CI) | |
| --- | --- | --- | --- | --- | --- |
|  |  | Normal weight | General obesity | Normal weight | General obesity |
| Total | | | | | |
|  | Crude model | 1.00 | 2.19 (1.57, 3.06) | 0.94 (0.61, 1.45) | 2.23 (1.54, 3.25) |
|  | Model 1 | 1.00 | 2.20 (1.57, 3.08) | 0.96 (0.62, 1.48) | 2.33 (1.59, 3.40) |
|  | Model 2 | 1.00 | 2.23 (1.59, 3.12) | 0.96 (0.62, 1.49) | 2.35 (1.61, 3.44) |
|  | Model 3 | 1.00 | 1.55 (1.06, 2.28) | 0.98 (0.63, 1.53) | 1.62 (1.06, 2.47) |
| Male | | | | | |
|  | Crude model | 1.00 | 2.73 (1.65, 4.53) | 0.67 (0.34, 1.32) | 3.12 (1.76, 5.52) |
|  | Model 1 | 1.00 | 2.74 (1.65, 4.57) | 0.67 (0.33, 1.33) | 3.16 (1.76, 5.66) |
|  | Model 2 | 1.00 | 2.81 (1.68, 4.68) | 0.68 (0.34, 1.36) | 3.24 (1.81, 5.83) |
|  | Model 3 | 1.00 | 1.62 (0.86, 3.04) | 0.66 (0.33, 1.32) | 1.84 (0.92, 3.68) |
| Female | | | | | |
|  | Crude model | 1.00 | 1.85 (1.19, 2.89) | 1.26 (0.72, 2.22) | 1.79 (1.09, 2.95) |
|  | Model 1 | 1.00 | 1.85 (1.18, 2.90) | 1.35 (0.76, 2.38) | 1.89 (1.14, 3.12) |
|  | Model 2 | 1.00 | 1.86 (1.19, 2.91) | 1.34 (0.76, 2.36) | 1.88 (1.14, 3.11) |
|  | Model 3 | 1.00 | 1.52 (0.94, 2.47) | 1.38 (0.78, 2.45) | 1.52 (0.88, 2.60) |
| Less Severely affected area | | | | | |
|  | Crude model | 1.00 | 2.41 (1.57, 3.70) | 0.96 (0.55, 1.66) | 2.48 (1.55, 3.96) |
|  | Model 1 | 1.00 | 2.42 (1.58, 3.73) | 1.01 (0.58, 1.75) | 2.58 (1.61, 4.15) |
|  | Model 2 | 1.00 | 2.44 (1.59, 3.76) | 1.01 (0.58, 1.75) | 2.60 (1.62, 4.19) |
|  | Model 3 | 1.00 | 1.64 (1.02, 2.66) | 1.07 (0.61, 1.88) | 1.75 (1.04, 2.94) |
| Severely affected area | | | | | |
|  | Crude model | 1.00 | 1.86 (1.09, 3.19) | 0.91 (0.45, 1.86) | 1.79 (0.94, 3.40) |
|  | Model 1 | 1.00 | 1.87 (1.09, 3.21) | 0.86 (0.42, 1.76) | 1.79 (0.93, 3.43) |
|  | Model 2 | 1.00 | 1.90 (1.11, 3.27) | 0.86 (0.42, 1.78) | 1.82 (0.95, 3.50) |
|  | Model 3 | 1.00 | 1.40 (0.74, 2.64) | 0.85 (0.41, 1.75) | 1.32 (0.63, 2.76) |

Note: OR, odds ratio; CI, confidence interval; Associations were expressed as odds ratio with 95% confidence interval; According to the cut-off points proposed by the Working Group on Obesity in China, general overweight/obesity was defined as BMI ≥ 24.0 kg/m^2^. Model 1 was adjusted for sex (not included if it was used as a stratification index), marital status, region, education, and famine severity (not included if it was used as a stratification index). Model 2 was additionally adjusted for smoking and drinking history. Model 3 was further adjusted for central obesity based on model 2.

**Table S5.** Associations of concurrent famine exposure and general obesity with T2DM in Comparison 2.

|  |  | Childhood-exposure  OR (95% CI) | | Fetal-exposure  OR (95% CI) | |
| --- | --- | --- | --- | --- | --- |
|  |  | Normal weight | General obesity | Normal weight | General obesity |
| Total | | | | | |
|  | Crude model | 1.00 | 1.83 (1.33, 2.52) | 0.95 (0.63, 1.42) | 2.58 (1.87, 3.56) |
|  | Model 1 | 1.00 | 1.74 (1.26, 2.40) | 0.94 (0.63, 1.41) | 2.50 (1.80, 3.47) |
|  | Model 2 | 1.00 | 1.75 (1.26, 2.41) | 0.94 (0.63, 1.41) | 2.51 (1.81, 3.49) |
|  | Model 3 | 1.00 | 1.34 (0.93, 1.95) | 0.93 (0.62, 1.39) | 1.96 (1.35, 2.86) |
| Male | | | | | |
|  | Crude model | 1.00 | 1.95 (1.20, 3.17) | 0.67 (0.34, 1.31) | 3.12 (1.90, 5.13) |
|  | Model 1 | 1.00 | 1.91 (1.16, 3.13) | 0.67 (0.34, 1.33) | 3.12 (1.87, 5.21) |
|  | Model 2 | 1.00 | 1.94 (1.18, 3.18) | 0.67 (0.34, 1.33) | 3.17 (1.90, 5.30) |
|  | Model 3 | 1.00 | 1.30 (0.70, 2.42) | 0.66 (0.33, 1.30) | 2.16 (1.14, 4.08) |
| Female | | | | | |
|  | Crude model | 1.00 | 1.69 (1.11, 2.58) | 1.15 (0.69, 1.92) | 2.18 (1.43, 3.34) |
|  | Model 1 | 1.00 | 1.62 (1.06, 2.48) | 1.14 (0.69, 1.91) | 2.17 (1.42, 3.34) |
|  | Model 2 | 1.00 | 1.60 (1.04, 2.44) | 1.17 (0.70, 1.95) | 2.18 (1.42, 3.35) |
|  | Model 3 | 1.00 | 1.39 (0.87, 2.22) | 1.16 (0.69, 1.95) | 1.92 (1.19, 3.07) |
| Less Severely affected area | | | | | |
|  | Crude model | 1.00 | 2.51 (1.60, 3.93) | 1.52 (0.90, 2.58) | 3.65 (2.34, 5.69) |
|  | Model 1 | 1.00 | 2.38 (1.51, 3.75) | 1.52 (0.90, 2.59) | 3.42 (2.17, 5.39) |
|  | Model 2 | 1.00 | 2.36 (1.50, 3.71) | 1.52 (0.90, 2.59) | 3.40 (2.16, 5.36) |
|  | Model 3 | 1.00 | 1.75 (1.05, 2.91) | 1.52 (0.89, 2.58) | 2.53 (1.52, 4.21) |
| Severely affected area | | | | | |
|  | Crude model | 1.00 | 1.34 (0.84, 2.15) | 0.50 (0.25, 1.00) | 1.79 (1.09, 2.97) |
|  | Model 1 | 1.00 | 1.25 (0.77, 2.03) | 0.50 (0.25, 1.01) | 1.79 (1.07, 2.99) |
|  | Model 2 | 1.00 | 1.28 (0.79, 2.07) | 0.49 (0.24, 0.98) | 1.82 (1.08, 3.04) |
|  | Model 3 | 1.00 | 0.97 (0.55, 1.71) | 0.47 (0.23, 0.94) | 1.37 (0.76, 2.49) |

Note: OR, odds ratio; CI, confidence interval; Associations were expressed as odds ratio with 95% confidence interval; According to the cut-off points proposed by the Working Group on Obesity in China, general overweight/obesity was defined as BMI ≥ 24.0 kg/m^2^. Model 1 was adjusted for sex (not included if it was used as a stratification index), marital status, region, education, and famine severity (not included if it was used as a stratification index). Model 2 was additionally adjusted for smoking and drinking history. Model 3 was further adjusted for central obesity based on model 2.

**Table S6.** Associations of concurrent famine exposure and central obesity with T2DM after excluding participants born in 1959 and 1961 from exposure group in Comparison 1.

|  | Non-exposure  OR (95% CI) | | | Fetal-exposure  OR (95% CI) | |
| --- | --- | --- | --- | --- | --- |
|  | Normal weight | Central obesity | Normal weight | | Central obesity |
| Total | | | | | |
| Crude model | 1.00 | 2.53 (1.69, 3.81) | 0.90 (0.46, 1.76) | | 2.64 (1.66, 4.20) |
| Model 1 | 1.00 | 2.70 (1.79, 4.08) | 0.91 (0.47, 1.79) | | 2.89 (1.80, 4.65) |
| Model 2 | 1.00 | 2.73 (1.81, 4.13) | 0.92 (0.47, 1.80) | | 2.92 (1.82, 4.70) |
| Model 3 | 1.00 | 1.95 (1.21, 3.14) | 0.95 (0.48, 1.87) | | 2.10 (1.24, 3.57) |
| Male | | | | | |
| Crude model | 1.00 | 3.53 (1.97, 6.32) | 0.75 (0.28, 1.98) | | 4.08 (2.09, 7.95) |
| Model 1 | 1.00 | 3.56 (1.98, 6.40) | 0.75 (0.28, 1.99) | | 4.12 (2.08, 8.15) |
| Model 2 | 1.00 | 3.60 (2.00, 6.49) | 0.76 (0.29, 2.01) | | 4.21 (2.12, 8.35) |
| Model 3 | 1.00 | 2.37 (1.14, 4.93) | 0.79 (0.30, 2.09) | | 2.90 (1.32, 6.36) |
| Female | | | | | |
| Crude model | 1.00 | 1.97 (1.11, 3.49) | 1.11 (0.44, 2.81) | | 1.89 (0.98, 3.62) |
| Model 1 | 1.00 | 1.97 (1.11, 3.49) | 1.18 (0.47, 3.02) | | 1.99 (1.03, 3.83) |
| Model 2 | 1.00 | 1.97 (1.11, 3.49) | 1.17 (0.46, 2.99) | | 2.00 (1.04, 3.85) |
| Model 3 | 1.00 | 1.50 (0.80, 2.83) | 1.22 (0.48, 3.11) | | 1.50 (0.73, 3.06) |
| Less Severely affected area | | | | | |
| Crude model | 1.00 | 2.81 (1.65, 4.79) | 1.01 (0.44, 2.32) | | 3.03 (1.67, 5.48) |
| Model 1 | 1.00 | 3.02 (1.76, 5.19) | 1.09 (0.47, 2.51) | | 3.44 (1.87, 6.32) |
| Model 2 | 1.00 | 3.03 (1.76, 5.21) | 1.09 (0.47, 2.51) | | 3.46 (1.88, 6.36) |
| Model 3 | 1.00 | 2.17 (1.17, 4.04) | 1.13 (0.49, 2.62) | | 2.48 (1.26, 4.89) |
| Severely affected area | | | | | |
| Crude model | 1.00 | 2.15 (1.14, 4.04) | 0.74 (0.23, 2.33) | | 2.02 (0.94, 4.36) |
| Model 1 | 1.00 | 2.33 (1.23, 4.43) | 0.67 (0.21, 2.14) | | 2.08 (0.95, 4.56) |
| Model 2 | 1.00 | 2.37 (1.25, 4.52) | 0.68 (0.21, 2.17) | | 2.09 (0.95, 4.60) |
| Model 3 | 1.00 | 1.75 (0.82, 3.77) | 0.71 (0.22, 2.28) | | 1.60 (0.67, 3.82) |

Note: OR, odds ratio; CI, confidence interval; Associations were expressed as odds ratio with 95% confidence interval; According to the cut-off points proposed by the Working Group on Obesity in China, central overweight/obesity was defined as waist circumference (WC) ≥ 85 cm in males or ≥ 80 cm in females. Model 1 was adjusted for sex (not included if it was used as a stratification index), marital status, region, education, and famine severity (not included if it was used as a stratification index). Model 2 was additionally adjusted for smoking and drinking history. Model 3 was further adjusted for general obesity based on model 2.

**Table S7.** Associations of concurrent famine exposure and central obesity with T2DM after excluding participants born in 1959 and 1961 from exposure group in Comparison 2.

|  | Non-exposure  OR (95% CI) | | | Fetal-exposure  OR (95% CI) | |
| --- | --- | --- | --- | --- | --- |
|  | Normal weight | Central obesity | Normal weight | | Central obesity |
| Total | | | | | |
| Crude model | 1.00 | 1.92 (1.35, 2.72) | 0.56 (0.27, 1.17) | | 2.80 (1.91, 4.12) |
| Model 1 | 1.00 | 1.77 (1.23, 2.53) | 0.54 (0.26, 1.12) | | 2.63 (1.77, 3.91) |
| Model 2 | 1.00 | 1.77 (1.24, 2.54) | 0.54 (0.26, 1.12) | | 2.64 (1.78, 3.93) |
| Model 3 | 1.00 | 1.34 (0.88, 2.04) | 0.55 (0.26, 1.14) | | 1.98 (1.25, 3.15) |
| Male | | | | | |
| Crude model | 1.00 | 2.07 (1.26, 3.41) | 0.47 (0.16, 1.36) | | 2.93 (1.64, 5.22) |
| Model 1 | 1.00 | 1.98 (1.19, 3.30) | 0.46 (0.16, 1.34) | | 2.90 (1.60, 5.25) |
| Model 2 | 1.00 | 1.97 (1.18, 3.29) | 0.46 (0.16, 1.35) | | 2.88 (1.59, 5.22) |
| Model 3 | 1.00 | 1.37 (0.71, 2.67) | 0.47 (0.16, 1.39) | | 2.02 (0.97, 4.22) |
| Female | | | | | |
| Crude model | 1.00 | 1.65 (1.00, 2.75) | 0.66 (0.24, 1.81) | | 2.45 (1.43, 4.21) |
| Model 1 | 1.00 | 1.59 (0.95, 2.64) | 0.62 (0.23, 1.72) | | 2.39 (1.39, 4.11) |
| Model 2 | 1.00 | 1.57 (0.94, 2.62) | 0.64 (0.23, 1.77) | | 2.44 (1.42, 4.21) |
| Model 3 | 1.00 | 1.26 (0.71, 2.23) | 0.64 (0.23, 1.78) | | 1.95 (1.06, 3.59) |
| Less Severely affected area | | | | | |
| Crude model | 1.00 | 2.28 (1.37, 3.78) | 0.88 (0.36, 2.11) | | 3.96 (2.33, 6.73) |
| Model 1 | 1.00 | 2.06 (1.23, 3.46) | 0.80 (0.33, 1.93) | | 3.51 (2.03, 6.06) |
| Model 2 | 1.00 | 2.06 (1.22, 3.46) | 0.79 (0.33, 1.93) | | 3.55 (2.05, 6.14) |
| Model 3 | 1.00 | 1.42 (0.78, 2.60) | 0.78 (0.32, 1.89) | | 2.38 (1.26, 4.50) |
| Severely affected area | | | | | |
| Crude model | 1.00 | 1.69 (1.03, 2.76) | 0.28 (0.06, 1.20) | | 1.84 (1.02, 3.33) |
| Model 1 | 1.00 | 1.63 (0.98, 2.72) | 0.28 (0.06, 1.20) | | 1.90 (1.03, 3.53) |
| Model 2 | 1.00 | 1.63 (0.97, 2.72) | 0.28 (0.06, 1.21) | | 1.82 (0.98, 3.38) |
| Model 3 | 1.00 | 1.42 (0.77, 2.62) | 0.28 (0.06, 1.21) | | 1.58 (0.78, 3.20) |

Note: OR, odds ratio; CI, confidence interval; Associations were expressed as odds ratio with 95% confidence interval; According to the cut-off points proposed by the Working Group on Obesity in China, central overweight/obesity was defined as waist circumference (WC) ≥ 85 cm in males or ≥ 80 cm in females. Model 1 was adjusted for sex (not included if it was used as a stratification index), marital status, region, education, and famine severity (not included if it was used as a stratification index). Model 2 was additionally adjusted for smoking and drinking history. Model 3 was further adjusted for general obesity based on model 2.
